# Diagnostic Significance of Trophoblast Cell Surface Antigen-2 Expression in Benign and Malignant Thyroid Lesions

**DOI:** 10.7759/cureus.97140

**Published:** 2025-11-18

**Authors:** Antony Queen Jeneffar, Sivaraman Anitharani

**Affiliations:** 1 Pathology, Government Kilpauk Medical College Hospital, Chennai, IND

**Keywords:** immunohistochemistry, papillary thyroid carcinoma, thyroid neoplasms, trophoblast cell surface antigen-2 (trop-2), tumour biomarkers

## Abstract

Introduction

Trophoblast cell surface antigen-2 (TROP-2), a transmembrane glycoprotein associated with trophoblast cells, is found to have a potential role in the proliferation of cancer cells and their ability to progress, invade and survive. An immunohistochemical study of TROP-2 can be helpful to differentiate benign and malignant thyroid lesions and guide clinical management. The study aimed to evaluate the diagnostic significance of TROP-2 expression in thyroid lesions.

Materials and methods

This cross-sectional study was conducted in a tertiary care teaching hospital, which included 44 cases of benign and malignant thyroid lesions. Tissue sections (4 µm thick) were obtained from paraffin-embedded blocks after deparaffinization and antigen retrieval in citrate buffer. The samples were tested for TROP-2 expression by immunohistochemistry. Collected data were entered in Microsoft Excel (Microsoft Corporation, Redmond, WA), and statistical analysis was performed using Statistical Packages for Social Sciences (SPSS) Version 23.0 (IBM Corp., Armonk, NY). A p-value less than 0.05 was considered statistically significant.

Results

The mean age of the study population was 42.84, and the range was from 16 years to 75 years. Among them, 37 subjects (84.09%) were female. On histopathological examination, 17 cases (38.64%) were diagnosed as benign thyroid lesions, 26 cases (59.09%) were of malignant thyroid lesions, and one case was diagnosed as a borderline lesion (non-invasive follicular thyroid neoplasm with papillary-like nuclear features (NIFTP)).

The proportion of TROP-2 positivity was significantly different between benign and malignant thyroid lesions, and between papillary thyroid carcinoma (PTC) and non-PTC malignant lesions. The sensitivity of TROP-2 IHC testing was found to be 69.23% for diagnosing malignant thyroid lesions and 78.26% for diagnosing PTC cases. The specificity and positive predictive value in both conditions were observed to be 100%. The proportion of TROP-2 positivity did not significantly differ among the different tumour stages and lymph node status groups.

Conclusion

TROP-2 as a diagnostic marker was observed to have high specificity, which indicates that it has a promising role as an adjunct diagnostic method to the currently available methods. However, TROP-2's sensitivity is found to be moderate, which indicates that this marker cannot be used as a standalone diagnostic tool but rather in conjunction with other markers and histopathological examination.

## Introduction

Thyroid carcinoma is the most common malignancy affecting the endocrine gland, and most cases are diagnosed at advanced stages. An increasing incidence of malignancy is noted among younger individuals. Papillary thyroid carcinoma (PTC) is the most common type of thyroid cancer, and the other types are follicular, medullary and anaplastic. These types, arising from thyroid follicular cells, are considered well-differentiated thyroid cancers. Poorly differentiated thyroid cancer, making up about 5%, and anaplastic thyroid cancer, about 1%, are more aggressive forms. Medullary thyroid cancer (MTC), which constitutes about 4%, originates from parafollicular C cells [1,2].

While early-stage thyroid cancers, which are usually well-differentiated, have excellent survival rates, advanced-stage cases like poorly differentiated and anaplastic thyroid cancers pose significant challenges [3,4]. MTC, although rare, is particularly deadly and often linked to germline RET mutations. There is a need for a multidisciplinary approach in managing advanced cases of thyroid malignancy, where surgery and radioactive iodine (RAI) may not be effective [5].

Immunohistochemistry (IHC) has a crucial role to play in thyroid pathology [6,7]. IHC could help in differentiating benign and malignant thyroid lesions, which guides clinical decision-making. TROP-2, a transmembrane glycoprotein associated with trophoblast cells, is found to have a potential role in the proliferation of cancer cells and their ability to progress, invade and survive. TROP-2 is observed to be overexpressed in many malignant conditions; hence, it has been evaluated by researchers as a marker in diagnosis as well as prognosis of cancer [8]. TROP-2 is also evaluated as a target for novel medicines, which can add a targeted approach towards only cancer cells without harming the normal cells. Antibody-drug conjugates, such as Sacituzumab Govitecan and Datopotamab Deruxtecan, are developed to target the cancer cells expressing TROP-2 [9].

Accurate diagnosis of PTC is challenging due to overlapping histological features with benign and other malignant thyroid conditions. Traditional histopathology often requires supplementary tools like IHC for ambiguous cases. CK19 is one of the commonly used IHC markers to detect PTC, but it has the drawback of having a higher number of false positives in benign thyroid conditions. Studies comparing TROP-2 with markers like HBME-1 and Galectin-3 suggest that TROP-2 offers higher sensitivity and specificity for PTC, particularly in distinguishing PTC from benign lesions and other neoplasms [10-12].

Research works have also been undertaken to explore the role of TROP-2 in determining the invasiveness of thyroid malignancy [13,14]. In studies assessing TROP-2’s diagnostic utility, it has displayed superiority over other markers, especially in the classical variant of papillary carcinoma. It can serve as a potential tool in diagnosing PTC, even in challenging situations with indeterminate tissue samples. Appropriate usage of this marker could improve the accuracy in diagnosing thyroid cases. The reliability and accuracy of TROP-2 in clinical practice have to be further validated, and studies have to be undertaken to explore the same. With this background, the study was conducted to assess the diagnostic utility of TROP-2 immunohistochemical expression in distinguishing benign from malignant thyroid lesions, with particular emphasis on PTC.

## Materials and methods

This study was done as a cross-sectional study in a government tertiary care teaching hospital. All cases reported as benign or malignant thyroid lesions by histopathological examination of thyroidectomy specimens received at the Department of Pathology during the period from January 2023 to June 2024 were included in the study.

Sample size estimation

Sample size estimation was done using nMaster software version 2.0 (Christian Medical College, Vellore, India). The sensitivity of TROP-2 was found to be 86.67% as per the study done by Khalil et al. [15].

 \[n = \frac{Z_{1-\alpha/2}^2 \, P \, (1-P)}{d^2}\] 

where P = sensitivity of TROP-2 (86.67%); d = precision (10); and Z = desired confidence level (95%). Using the formula, the sample size was calculated as 44. 

Data collection and methods

A total of 44 cases were included in the study, as determined by the calculated sample size. Approval from the Institutional Ethics Committee was sought before initiating the study. Clinical and relevant demographic details were collected from the patient records while receiving the thyroidectomy specimen for histological examination. Two pathologists reviewed all cases to establish the histological type and characteristics of benign and malignant thyroid lesions according to WHO criteria.

The specimens were fixed in 10% neutral buffer formalin, and tissue samples were submitted for routine Haematoxylin and Eosin staining. Parameters such as the tumour's focality, size, histological type, stage, and nodal status were observed and documented.

Tissue sections (4 µm thick) were obtained from paraffin-embedded blocks after deparaffinization and antigen retrieval in citrate buffer. Recombinant TROP-2 rabbit monoclonal antibody was obtained from Invitrogen (Thermo Fisher Scientific, Waltham, MA). Antibody dilution was prepared as per the manufacturer's instructions. Diluted TROP-2 antibody was applied to the tissue sections and incubated for 60 minutes at room temperature. Slides were counterstained with haematoxylin for one minute and mounted. Appropriate positive and negative controls were included [16]. Membranous staining of tumour cells was observed. Moderate (2+) and strong (3+) staining intensities were considered positive for TROP-2 expression.

Data analysis

Data collection was done and entered into Microsoft Excel (Microsoft Corporation, Redmond, WA). Statistical analysis was performed using Statistical Packages for Social Sciences (SPSS) Version 23.0 (IBM Corp., Armonk, NY). Mean, median, range, and proportion were used to express central tendency. Standard deviation was used to express data dispersion. Fischer's exact test or chi-square test was used to compare data expressed as proportions. An unpaired t-test was used to compare the means between groups. A p-value less than 0.05 was considered statistically significant. Graphical data were drawn using Microsoft Excel.

## Results

The clinicopathological characteristics of the study subjects were tabulated in Table 1. A total of 44 cases were included in the study. The mean age of the study population was 42.84, and the range was from 16 years to 75 years. Most of the subjects were between 31 and 50 years of age (22 cases, 50%). Nine of the patients were below 30 years of age (nine cases, 20.4%). The mean age of benign and malignant thyroid cases was 44.0 (14.18) and 42.04 (15.06), respectively, and the proportion of female gender was 94.4 and 76.9%, which were compared and found to be insignificant. Clinicopathological characteristics of the PTC cases were tabulated in Table 2. The classical variant was the most common type noted (14 of 23 cases; 60.87%). All the cases were of a unifocal nature. Most of the cases belonged to tumour stage 2 (13; 56.5%). In most cases, the lymph node status could not be determined (13; 56.5%).

**Table 1:** Clinicopathological characteristics of study population NIFTP - non-invasive follicular thyroid neoplasm with papillary-like nuclear features

S. No.	Variable		N = 44	Percentage (%)
1	Age (years)	Mean (SD)	42.84 (14.57)	-
		Median	41.5	-
		Range	16-75	-
2	Gender	Female	37	84.09
		Male	7	15.91
3	Type of surgery	Total thyroidectomy	27	61.36
		Hemithyroidectomy	17	38.64
4	Histopathological diagnosis	Benign thyroid lesions	17	38.64
		Borderline - NIFTP	1	2.27
		Malignant thyroid lesions	26	59.09
5	Benign thyroid lesions	Follicular adenoma	5	11.36
		Multinodular goitre	8	18.18
		Hürthle cell adenoma	2	4.54
		Hashimoto’s thyroiditis	1	2.27
		Granulomatous thyroiditis	1	2.27
6	Malignant thyroid lesions	Papillary carcinoma - classical variant	14	31.81
		Papillary carcinoma - follicular variant	5	11.36
		Papillary microcarcinoma	4	9.09
		Follicular carcinoma	2	4.54
		Medullary carcinoma	1	2.27

**Table 2:** Clinicopathological characteristics of papillary thyroid carcinoma cases

S. No.	Variable		N = 23	Percentage (%)
1	Age (years)	Mean (SD)	40.61 (15.19)	-
		Median	39	-
		Range	16 - 75	-
2	Gender	Female	18	78.3
		Male	5	21.7
3	Type of surgery	Total thyroidectomy	16	69.6
		Hemithyroidectomy	7	30.4
4	Histopathological type	Papillary carcinoma - classical variant	14	60.87
		Papillary carcinoma - follicular variant	5	21.74
		Papillary microcarcinoma	4	17.39
5	T stage	T1	7	30.4
		T2	13	56.5
		T3	2	8.7
		T4	1	4.3
6	Lymph node status	Positive lymph node (N1)	5	21.7
		Negative lymph node (N0)	5	21.7
		Unknown (Nx)	13	56.5
7	Focality	Unifocal	23	100
		Multifocal	0	0

**Figure 1:**
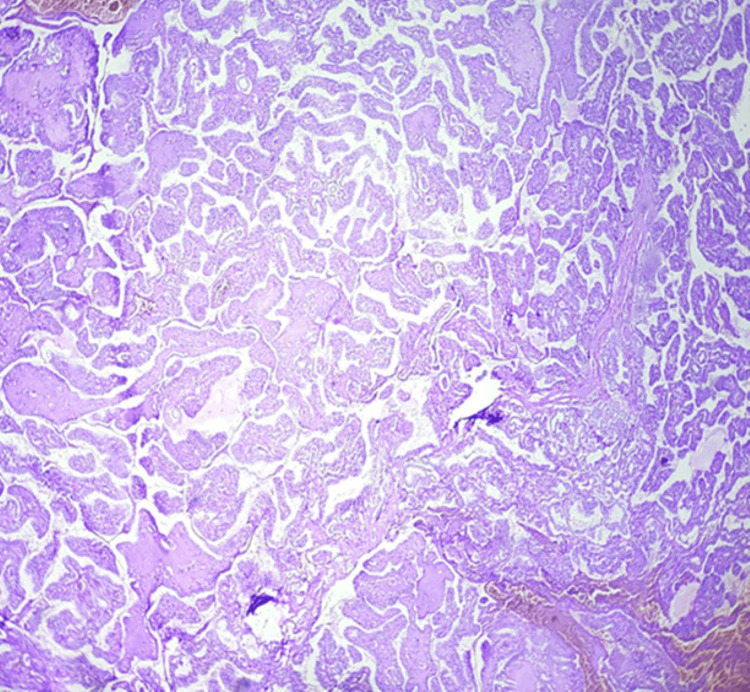
Papillary carcinoma showing branching papillae with hyalinized core (40×)

**Figure 2:**
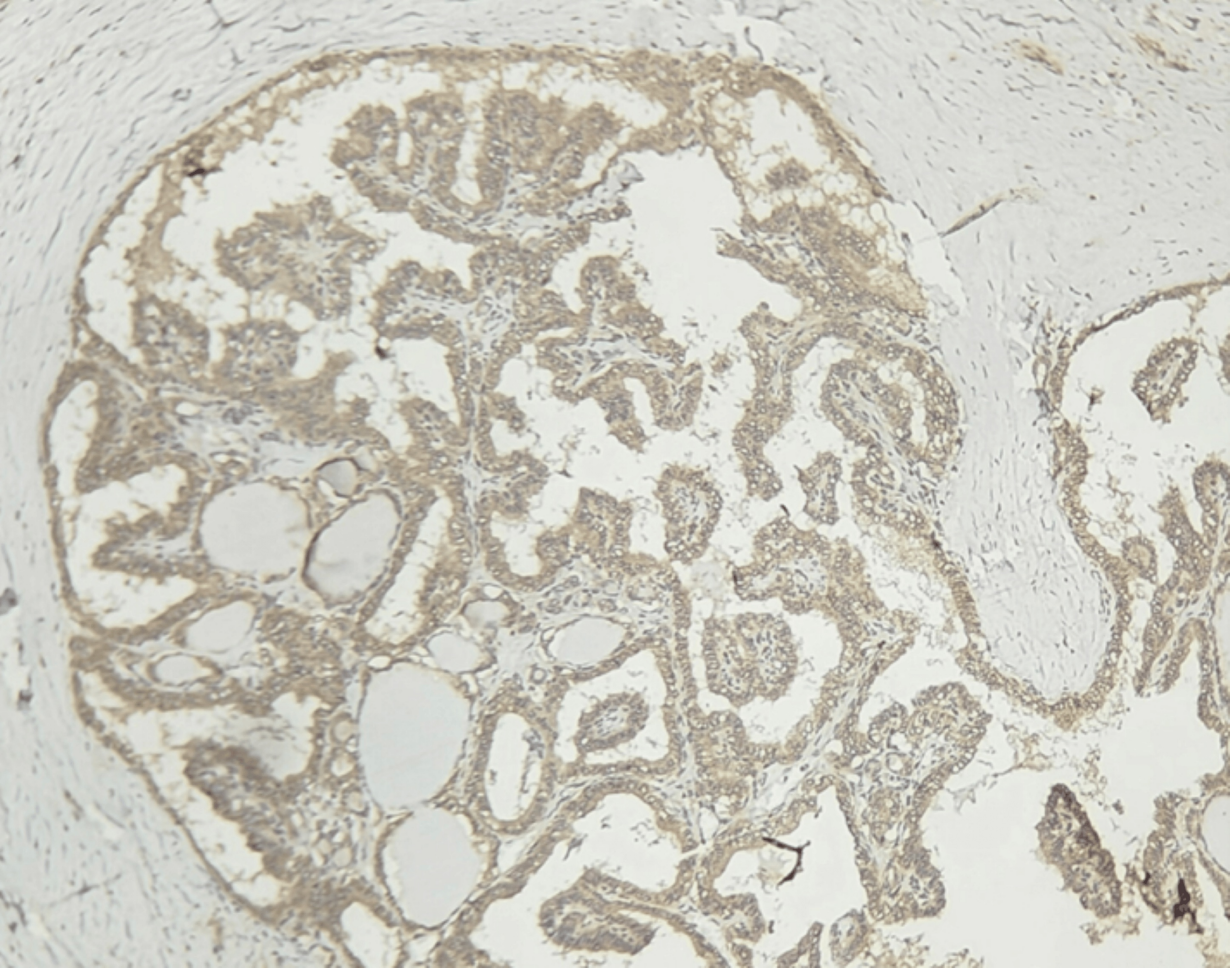
Papillary carcinoma showing TROP-2 positivity (100×)

**Figure 3:**
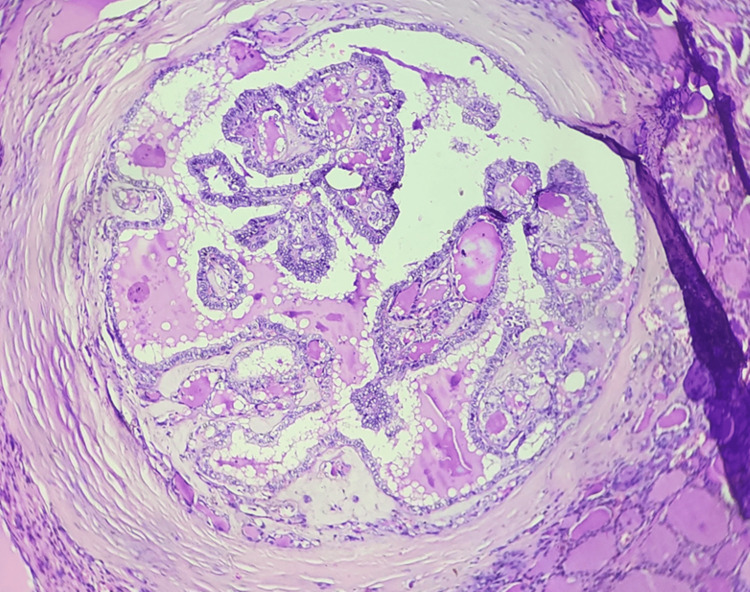
Microcarcinoma - papillary, classic subtype (100×)

**Figure 4:**
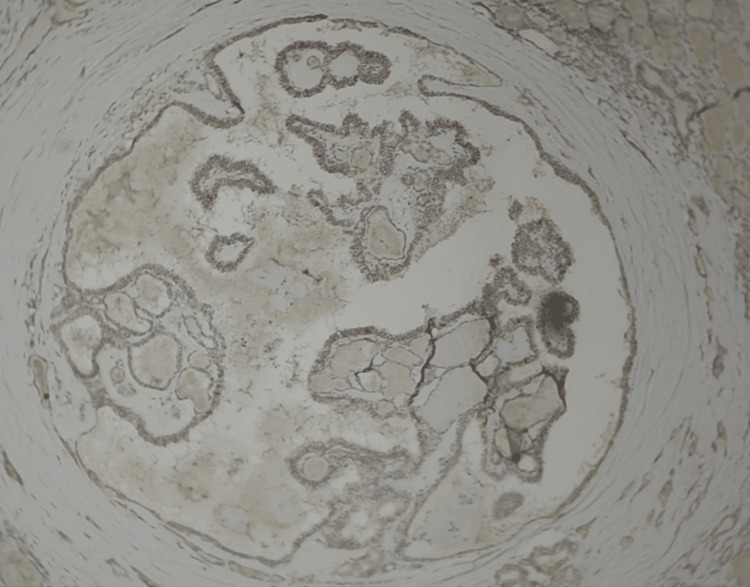
Microcarcinoma, papillary type showing TROP-2 positivity (100×)

**Figure 5:**
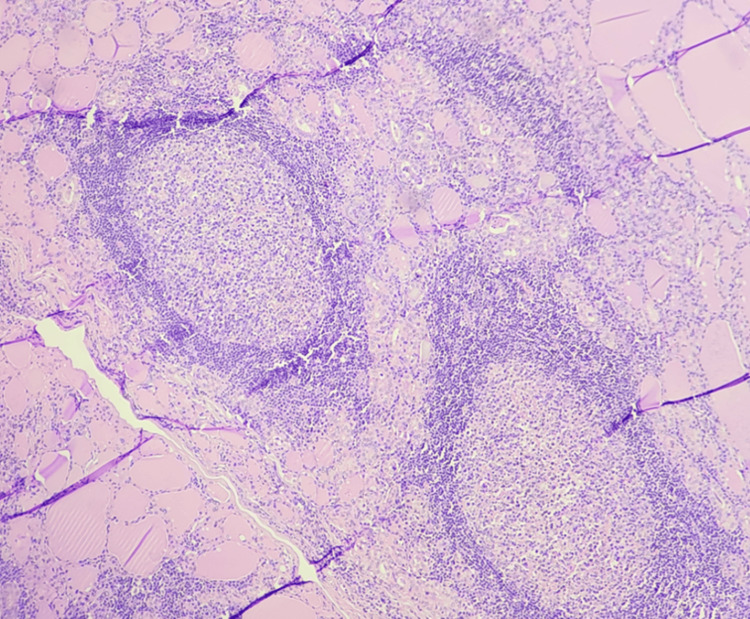
Hashimoto's thyroiditis (100×)

**Figure 6:**
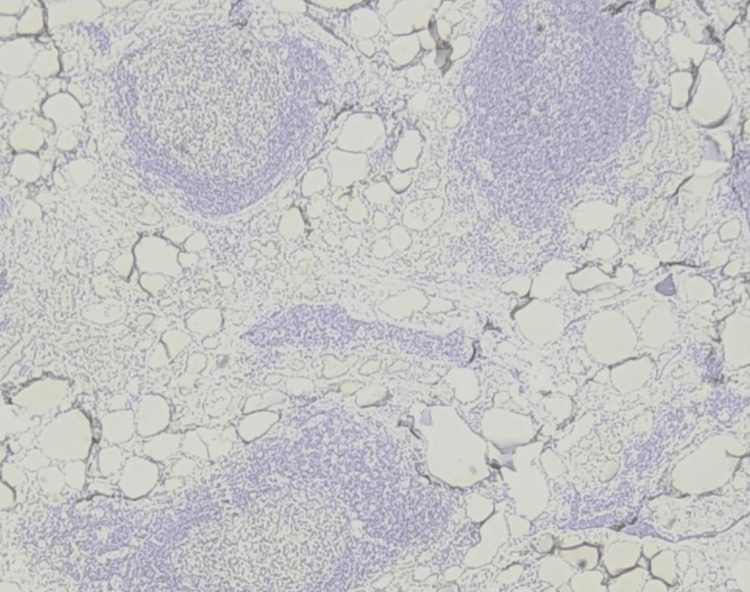
TROP-2 negativity in Hashimoto’s thyroiditis (100×)

TROP-2 positivity among the benign, borderline, and malignant thyroid lesions was tabulated in Table 3 (Figures 1-4). None of the benign cases were TROP-2 positive in IHC staining (Figures 5-6). Of the 23 PTC cases, 18 were TROP-2 positive (18 of 23; 78.26%). Among the PTC variants, the classical variant had a TROP-2 positivity proportion of 92.9%, followed by the microcarcinoma variant (three of four cases; 75%). The thyroid follicular lesions had only a 10% TROP-2 positivity proportion (two cases out of 20). The proportion of TROP-2 positivity did not differ significantly among the different tumour stages and lymph node status groups (Table 4).

**Table 3:** TROP-2 expression in benign and malignant thyroid lesions NIFTP - non-invasive follicular thyroid neoplasm with papillary-like nuclear features

S. No.	Diagnosis	Number of Cases (N = 44)	TROP-2 Positive	TROP-2 Negative	Positive Percentage (%)
1	Follicular adenoma	5	0	5	0
2	Multinodular goitre	8	0	8	0
3	Hürthle cell adenoma	2	0	2	0
4	Hashimoto’s thyroiditis	1	0	1	0
5	Granulomatous thyroiditis	1	0	1	0
6	Borderline - NIFTP	1	0	1	0
7	Papillary carcinoma - classical variant	14	13	1	92.9
8	Papillary carcinoma - follicular variant	5	2	3	40
9	Papillary microcarcinoma	4	3	1	75
10	Follicular carcinoma	2	0	2	0
11	Medullary carcinoma	1	0	1	0
Total		44	18	26	40.9

**Table 4:** Comparison of tumour stage (proportion), lymph node status (proportion) and TROP-2 positivity *Fisher’s exact test: Comparison of TROP-2 positivity between lymph node positive vs negative cases. p-value: 0.4545 (not significant).

Tumour Stage/Lymph Node Status	TROP-2 Positive (%)	TROP-2 Negative	Total	Fisher’s Exact Value	p-value
Tumour stage					
T1	6 (66.7)	3	9	0.843	1.000
T2	9 (69.2)	4	13		
T3	2 (66.7)	1	3		
T4	1 (100)	0	1		
Lymph node status					
Positive	5 (100) *	0	5	2.637	0.315
Negative	4 (66.7) *	2	6		
Unknown	9 (60)	6	15		

**Figure 7:**
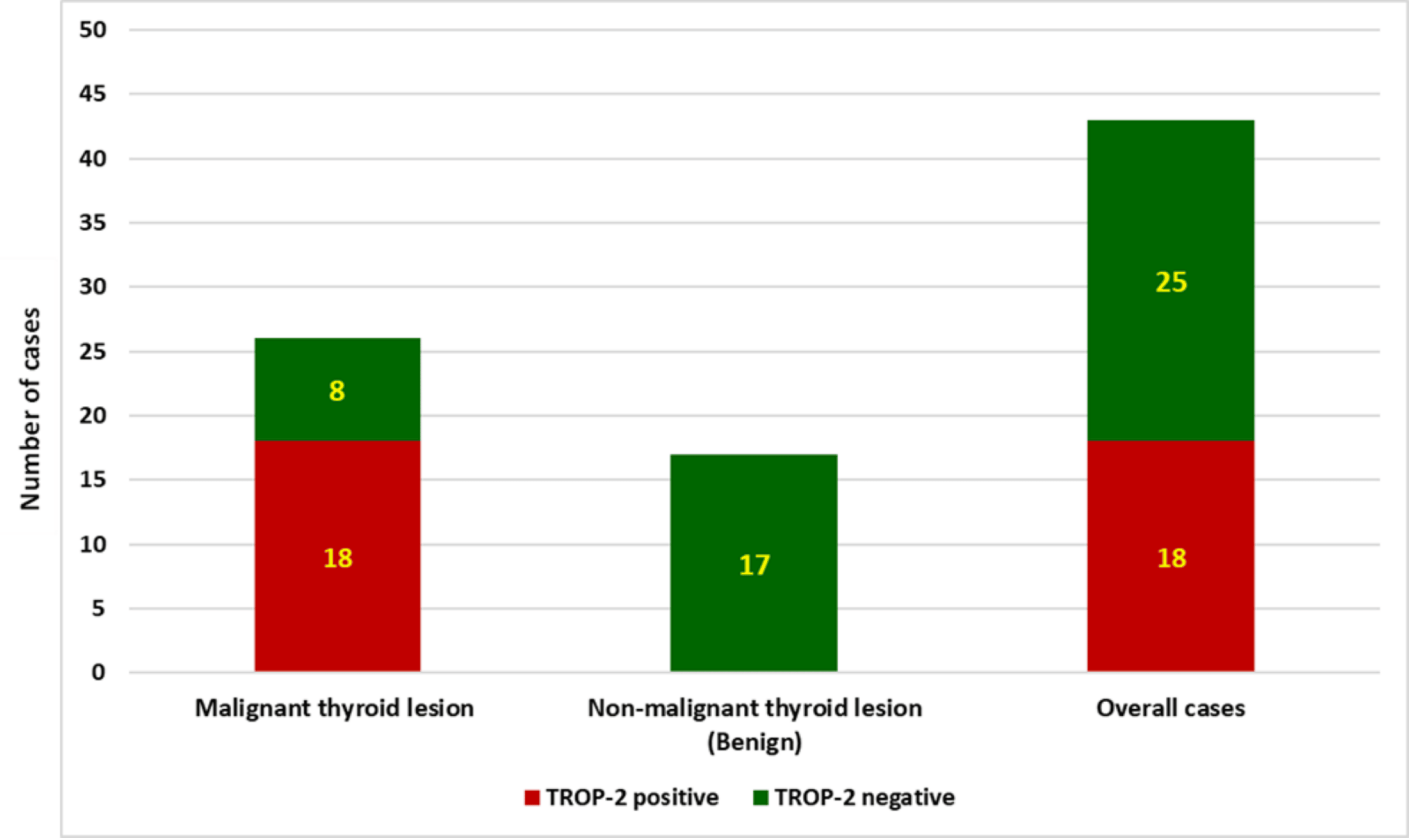
TROP-2 positivity among benign vs malignant thyroid lesions X-axis: type of thyroid lesion; Y-axis: number of cases

The proportion of TROP-2 positivity was significantly different between benign and malignant thyroid lesions and between PTC and non-PTC malignant lesions (Figure 7; Tables 5-6). The sensitivity of TROP-2 IHC testing was found to be 69.23% for diagnosing malignant thyroid lesions and 78.26% for diagnosing PTC cases. The specificity and positive predictive value in both conditions were observed to be 100%.

**Table 5:** Sensitivity, specificity, PPV, and NPV of benign vs malignant thyroid lesions for TROP-2 marker NIFTP case (borderline) was not included in the analysis (N = 43). Fisher’s exact test: p-value < 0.0001 (comparison of TROP-2 positivity percentage between malignant and benign thyroid lesions). NIFTP - non-invasive follicular thyroid neoplasm with papillary-like nuclear features; NPV - negative predictive value; PPV - positive predictive value

	TROP-2 Positive	TROP-2 Negative	Total
Malignant thyroid lesion	18	8	26
Non-malignant thyroid lesion (benign)	0	17	17
Total	18	25	43
Statistic	Value (%)	95% confidence interval (%)	
Sensitivity	69.23	48.21-85.67	
Specificity	100	80.49-100	
Positive predictive value	100	81.47-100	
Negative predictive value	68.0	54.42-79.09	

**Table 6:** Diagnostic performance of TROP-2 in differentiating papillary thyroid carcinoma from other thyroid malignant lesions Fisher’s exact test: p-value 0.0215 (comparison of proportion of TROP-2 cases between papillary and non-papillary thyroid cancer).

	TROP-2 Positive	TROP-2 Negative	Total
PTC lesions	18	5	23
Non-PTC malignant lesions	0	3	3
Total	18	8	26
Statistic	Value (%)	95% confidence interval (%)	
Sensitivity	78.26	56.30-92.54	
Specificity	100	29.24-100.0	
Positive predictive value	100	81.47-100.0	
Negative predictive value	37.50	21.65-56.58	

## Discussion

Thyroid carcinoma is one of the most common malignancies observed worldwide across countries. The incidence rate among women is three times higher than among men. Recent observations suggest that there is a worrisome increase in the incidence of thyroid cancer in younger age groups of both genders [17,18]. Various molecular markers are explored and studied in thyroid malignancy, which can help in early diagnosis and the timely selection of appropriate treatment modalities. We have undertaken this research work to evaluate the expression of the marker trophoblast cell surface antigen-2 (TROP-2) in benign and malignant thyroid lesions and assess its sensitivity and specificity.

TROP-2 is an important signalling molecule that interacts with ligands such as claudin-1 and 7, cyclin D1, and IGF-1. Through these interactions, TROP-2 is involved in regulating cell growth, proliferation, regeneration, and tumour cell proliferation, invasion and migration. As a potential modulator/enhancer of EpCAM-induced signalling, TROP-2 can prompt EpCAM proliferation and migration into the liver parenchyma [19]. Furthermore, TROP-2 facilitates cell migration even in the absence of growth factors, with induced foci formation signifying a loss of the ability to maintain cell growth and movement [20].

TROP-2 serves as a prognostic factor and marker for various cancers, showing overexpression in solid tumour cancers like breast, cervix, colorectal, oesophagus, gastric, lung, oral cavity squamous cell carcinoma, ovary, pancreas, prostate, stomach, thyroid, urinary bladder, and uterus. However, it is underexpressed in non-small lung cancer. Hematologic malignancies, including leukaemia, ENK/TL, and NHL, exhibit upregulated TROP-2, while anaplastic large cell lymphoma (ALCL) shows no TROP-2 expression [21]. Researchers are currently exploring antibodies, antibody-drug conjugates, and inhibitors to target TROP-2 expression, aiming to decrease its overexpression and curb tumour progression in specific cancers [9,22].

We included 44 cases of thyroid lesions during the study period; among them, 26 cases were diagnosed as malignant in histopathological examination. Among the malignant cases, PTC was found to be the predominant type. The age comparison between the benign and malignant cases (benign 44.0 years vs malignant 42.04 years) did not display a statistical difference. The mean age of the PTC classical variant (most common) was found to be 39.43 years, which is in line with the recent trend of malignant thyroid cases in the younger population.

Overall, a female preponderance was noted (37 out of 44 cases, 84.09%) among the study population. The female gender proportion in benign and malignant groups (94.4% in benign vs 76.9% in malignant) was significantly different, but again reinstated the female preponderance. This observation reinforces the epidemiological observation of higher benign as well as malignant thyroid disease among females.

Among the cases studied, 23 cases were PTC, the most common histopathological type of thyroid carcinoma. Numerous histopathological subtypes of PTC have been reported in the literature. They are conventional or classical, follicular variant, papillary microcarcinoma, tall cell, oncocytic, columnar cell, diffuse sclerosing, solid, clear cell, macrofollicular and others. Each variant was described to have a specific growth pattern, cell types, and stromal changes. However, uniform criteria to define these subtypes are lacking. In our study, the classical variant (60.87%), follicular variant (21.74%), and microcarcinoma (17.39%) were the subtypes observed. The mean age of PTC cases was 40.61 years, the female proportion was 78.3%, and the predominant subtype was the classical or conventional variant, which concurs with the scientific reports.

Regarding the tumour staging of papillary carcinoma cases, most of them were classified as T2 (56.5%) or T1 (30.4%). Cases belonging to advanced stages were far fewer (T3: 8.7%, T4: 4.3%). This observation underscores the detection of PTC cases in earlier stages due to the widespread use of imaging methods and fine-needle aspiration cytology (FNAC) study of thyroid nodules. Lymphatic spread is commonly observed in PTC cases, and cervical lymph nodes are commonly involved. Based on the extent of cervical lymph node involvement, surgical removal as a central or lateral lymph node dissection would be preferred. Distant metastasis is relatively rare (2-3%) in PTC cases. In our study, lymph node metastasis was noted in 21.7% of the cases, which is consistent with the known association between PTC and lymphatic spread. However, in 56.5% of the cases, the lymph node status was not assessed, possibly due to the absence of a comprehensive lymph node dissection during surgery. The observation highlighted the importance of preoperative and intraoperative lymph node assessment for staging, prevention of recurrence, and improvement of the prognosis among PTC patients.

Apart from categories of benign and malignant lesions in the thyroid, some cases are also categorised as borderline. The terms borderline and precursor lesions are used interchangeably many times. However, the term precursor lesions can also include benign, borderline, and low-malignant thyroid lesions. Indolent lesion of epithelial (IDLE) origin is also a term used to represent borderline or precursor lesions. Follicular neoplasm of uncertain malignant potential (FN-UMP), well-differentiated tumour of uncertain malignant potential (WDT-UMP), non-invasive follicular thyroid neoplasm with papillary-like nuclear features (NIFTP), and atypia of undetermined significance/follicular lesion of undetermined significance (AUS/FLUS) are some of the lesions categorised as borderline in thyroid cases. In our study, only one case of borderline lesion was noted, which was classified as NIFTP. It was previously considered to be a subtype of PTC, which displays PTC-like nuclear features but without lymphatic or vascular invasive characteristics [6].

We have assessed the diagnostic performance of TROP-2 testing to identify malignant lesions of the thyroid (Table 5). The sensitivity, specificity, positive predictive value (PPV), and negative predictive value (NPV) parameters were 69.23%, 100%, 100%, and 68%, respectively. In distinguishing benign and malignant thyroid lesions, TROP-2 testing yielded high specificity (100%) and PPV (100%) but moderate sensitivity (69.23%), which indicates that the probability of having malignancy is 100% when an individual is positive for TROP-2. NPV was 68%, meaning that in 32% of cases, a negative TROP-2 result could still be associated with malignancy.

We also assessed the performance of TROP-2 as a diagnostic test to distinguish PTC from non-papillary malignant lesions (Table 6). The sensitivity, specificity, PPV, and NPV parameters were 78.26%, 100%, 100%, and 37.5%, respectively. Among the malignant thyroid lesions, the probability of having PTC is 100% when a person tests positive for TROP-2. However, the NPV was low (37.5%), which means that TROP-2 negativity will not reliably rule out non-papillary malignant lesions.

The potential of TROP-2 as a diagnostic marker for PTC and to differentiate benign, malignant lesions has been explored in various studies (Table 7) [10-16,23-29]. Studies have compared the diagnostic performance of TROP-2 with other markers, such as HBME-1 and Galectin-3. TROP-2's role in thyroid cancer invasiveness has been investigated by correlating TROP-2 marker's expression with advanced tumour stages and lymph node involvement [13,14]. Establishing TROP-2's specificity, sensitivity, and overall accuracy could support its use as a reliable marker for routine diagnostic practice, improving the accuracy of thyroid cancer diagnosis and potentially reducing indeterminate cases.

**Table 7:** Summary of studies on TROP-2 as a diagnostic marker for papillary thyroid carcinoma (PTC) [10-16,23-29] cv - classical variant; FNA - fine-needle aspiration; fvPTC - follicular variant papillary thyroid carcinoma; IHC - immunohistochemistry; mPTC - micro-papillary thyroid carcinoma; NIFTP - non-invasive follicular thyroid neoplasm with papillary-like nuclear features; PTC - papillary thyroid carcinoma; TMA - tissue microarray; tcv - tall cell variant

Published Study	Study Focus	Study Sample	Key Findings on TROP-2
Bychkov et al. (2016) [10]	TROP-2 in PTC vs benign lesions	226 tumours, 207 controls	PTC cv, tcv and mPTC: diffuse positivity; fvPTC: focal; Sensitivity 98.1%, Specificity 97.5% for PTC cv
Liu et al. (2017) [11]	Expression in benign vs malignant lesions	Thyroid lesions	PTC: high TROP-2 positivity (94% classical, 84% fvPTC); benign: negative
Sun et al. (2021) [12]	Mechanistic study of TROP2 in PTC	60 PTC + adjacent tissue	High TROP2 in PTC linked to lymph node metastasis, size, capsular invasion
Abdou et al. (2019) [13]	TROP-2 vs CK19 in PTC and lymph node mets	FNA and surgical samples	High specificity for PTC; predictive for lymph node metastasis
Kılınc et al. (2022) [14]	TROP2 and tumour aggressiveness	270 thyroid cases, including 145 malignant and 124 benign thyroid cases	TROP2 expression was significantly correlated with older age, absence of encapsulation, infiltrative spreading, perineural invasion, extrathyroidal extension, and tall cell and/or hobnail differentiation
Khalil et al. (2020) [15]	TROP-2 in PTC and borderline lesions	55 thyroid cases	Sensitivity 86.7%, specificity 93.8%; borderline lesions show partial positivity
Addati et al. (2015) [16]	TROP-2 vs HBME-1 in cytological and histological samples	22 FNA samples + histology	Sensitivity 100%, specificity 89% in cytological samples, sensitivity 87% and specificity 89% in histological samples; concordance between immunocytochemistry (ICC) and IHC was 76% for HBME-1 and 91% for TROP-2
Simms et al. (2016) [23]	Diagnostic utility in FNA and tissue microarray (TMA) samples	FNA and TMA samples	Sensitivity: 95.3% (FNA), 90% (TMA); Specificity: 89% (FNA), 95.2% (TMA)
Murtezaoglu and Gucer (2017) [24]	TROP-2 vs HBME-1, Galectin-3, CK19	Benign vs malignant thyroid	Significant differential expression; combination of markers enhances accuracy
Sadullahoğlu et al. (2019) [25]	TROP-2 in malignancy	Malignant and benign cases	74.6% malignant TROP-2+, 89.5% in PTC; only 1.3% in benign lesions
Zargari and Mokhtari (2019) [26]	TROP-2 vs HBME-1 IHC performance	Thyroid lesions	TROP-2: sensitivity 93%, specificity 74%; HBME-1: sensitivity 84%, specificity 98%; dual-marker use improves diagnostic yield
Eid and Safia (2021) [27]	TROP-2 vs CK19 in 249 lesions	249 lesions	TROP-2: sensitivity 87.78%, specificity 100%; CK19: sensitivity 100%, specificity 25.69%
El-Dakrany et al. (2021) [28]	TROP2 and c-Kit in thyroid neoplasms	85 cases	TROP2+: 87.5% of PTC; not expressed in benign/NIFTP
Abu-Seadah et al. (2023) [29]	TROP-2 and HBME-1 correlation	50 specimens	TROP-2: 87.1% in malignant, 21.1% in benign; significant correlation with HBME-1

Study limitations

Due to the short study duration and small sample size, we were unable to include the full spectrum of benign, borderline, and malignant thyroid lesions. Inclusion of a wider range of cases would have better demonstrated the diagnostic significance of TROP-2 expression across various thyroid lesion types. Our analysis was confined to post-thyroidectomy specimens; incorporating pre-operative samples could have provided additional insight into the potential utility of TROP-2 during initial diagnostic workup. Furthermore, the study focused solely on TROP-2 IHC, as other established thyroid markers, such as CK19 and HBME-1, could not be evaluated due to resource and time constraints.

Implications and future directions

The observations from our study underscore the potential role of TROP-2 as a diagnostic marker to differentiate benign and malignant thyroid lesions, particularly in PTC. The marker is observed to have high specificity, which indicates that it has a promising role as an adjunct diagnostic method to the currently available methods, such as FNAC, which can yield inconclusive results in follicular patterned lesions. However, the sensitivity of TROP-2 is found to be moderate, which indicates that this marker cannot be used as a standalone diagnostic tool but rather should be used in conjunction with other markers and histopathological examination.

Further studies with larger sample sizes are required so that an adequate number of cases belonging to every subtype of thyroid lesion can be evaluated and the present study's findings can be validated. Long-term follow-up of cases for prognosis may help in assessing the potential role of TROP-2 and its implications as a prognostic marker. Further research can be undertaken to deeply investigate the mechanisms behind the increased expression of TROP-2 in thyroid malignancy, which can bring out the role of this marker in tumour development and progression into the spotlight.

## Conclusions

The proportion of TROP-2 positivity was significantly different between benign and malignant thyroid lesions and between PTC and non-PTC malignant lesions. The sensitivity of TROP-2 IHC testing was found to be 69.23% for diagnosing malignant thyroid lesions and 78.36% for diagnosing PTC cases. The specificity and positive predictive value in both conditions were observed to be 100%. TROP-2 as a diagnostic marker was observed to have high specificity, which indicates that it has a promising role as an adjunct diagnostic method to the currently available methods.

## References

[1] Bai Y, Kakudo K, Jung CK (2020). Updates in the pathologic classification of thyroid neoplasms: a review of the World Health Organization classification. Endocrinol Metab (Seoul).

[2] Haugen BR, Alexander EK, Bible KC (2016). 2015 American Thyroid Association management guidelines for adult patients with thyroid nodules and differentiated thyroid cancer: the American Thyroid Association guidelines task force on thyroid nodules and differentiated thyroid cancer. Thyroid.

[3] Alhejaily AG, Alhuzim O, Alwelaie Y (2023). Anaplastic thyroid cancer: pathogenesis, prognostic factors and genetic landscape (Review). Mol Clin Oncol.

[4] Menon G, Kashyap S, Naing PT (2025). Anaplastic thyroid cancer. StatPearls.

[5] Schlumberger M, Leboulleux S (2021). Current practice in patients with differentiated thyroid cancer. Nat Rev Endocrinol.

[6] Baloch Z, Mete O, Asa SL (2018). Immunohistochemical biomarkers in thyroid pathology. Endocr Pathol.

[7] Crescenzi A, Baloch Z (2023). Immunohistochemistry in the pathologic diagnosis and management of thyroid neoplasms. Front Endocrinol (Lausanne).

[8] Lombardi P, Filetti M, Falcone R (2023). Overview of Trop-2 in cancer: from pre-clinical studies to future directions in clinical settings. Cancers (Basel).

[9] Goldenberg DM, Cardillo TM, Govindan SV, Rossi EA, Sharkey RM (2015). Trop-2 is a novel target for solid cancer therapy with sacituzumab govitecan (IMMU-132), an antibody-drug conjugate (ADC). Oncotarget.

[10] Bychkov A, Sampatanukul P, Shuangshoti S, Keelawat S (2016). TROP-2 immunohistochemistry: a highly accurate method in the differential diagnosis of papillary thyroid carcinoma. Pathology.

[11] Liu H, Shi J, Lin F (2017). The potential diagnostic utility of TROP-2 in thyroid neoplasms. Appl Immunohistochem Mol Morphol.

[12] Sun H, Chen Q, Liu W (2021). TROP2 modulates the progression in papillary thyroid carcinoma. J Cancer.

[13] Abdou AG, Shabaan M, Abdallha R, Nabil N (2019). Diagnostic value of TROP-2 and CK19 expression in papillary thyroid carcinoma in both surgical and cytological specimens. Clin Pathol.

[14] Kılınc E, Gunes P, Doganer A (2022). TROP2 is a good indicator for infiltrative nature of carcinoma rather than diagnosing malignancy in thyroid. Indian J Otolaryngol Head Neck Surg.

[15] Khalil RM, Zaki SS, Farid RM (2020). The potential diagnostic value of TROP-2 expression in papillary thyroid carcinoma, benign thyroid lesions, and borderline thyroid lesions: immunohistochemical study. Egypt J Pathol.

[16] Addati T, Achille G, Centrone M (2015). TROP-2 expression in papillary thyroid cancer: a preliminary cyto-histological study. Cytopathology.

[17] Lim H, Devesa SS, Sosa JA, Check D, Kitahara CM (2017). Trends in thyroid cancer incidence and mortality in the United States, 1974-2013. JAMA.

[18] Kitahara CM, Sosa JA (2016). The changing incidence of thyroid cancer. Nat Rev Endocrinol.

[19] Szabo R, Ward JM, Artunc F, Bugge TH (2022). EPCAM and TROP2 share a role in claudin stabilization and development of intestinal and extraintestinal epithelia in mice. Biol Open.

[20] Nikiforov YE, Nikiforova MN (2011). Molecular genetics and diagnosis of thyroid cancer. Nat Rev Endocrinol.

[21] Goldenberg DM, Stein R, Sharkey RM (2018). The emergence of trophoblast cell-surface antigen 2 (TROP-2) as a novel cancer target. Oncotarget.

[22] Shastry M, Jacob S, Rugo HS, Hamilton E (2022). Antibody-drug conjugates targeting TROP-2: clinical development in metastatic breast cancer. Breast.

[23] Simms A, Jacob RP, Cohen C, Siddiqui MT (2016). TROP-2 expression in papillary thyroid carcinoma: potential diagnostic utility. Diagn Cytopathol.

[24] Murtezaoglu AR, Gucer H (2017). Diagnostic value of TROP-2 expression in papillary thyroid carcinoma and comparison with HBME-1, galectin-3 and cytokeratin 19. Pol J Pathol.

[25] Sadullahoğlu C, Sayıner A, Süren D, Yıldırım HT, Nergiz D, Sezer C, Oruç MT (2019). The diagnostic significance of trophoblast cell-surface antigen-2 expression in benign and malignant thyroid lesions. Indian J Pathol Microbiol.

[26] Zargari N, Mokhtari M (2019). Evaluation of diagnostic utility of immunohistochemistry markers of TROP-2 and HBME-1 in the diagnosis of thyroid carcinoma. Eur Thyroid J.

[27] Eid A, Abo Safia H (2021). TROP- 2: a unique immunohistochemical marker for diagnosis of papillary thyroid carcinoma. Egypt J Cancer Biomed Res.

[28] El-Dakrany AAE-H, Zamzam YAE-M, Wasfy RE (2021). The potential diagnostic utility of TROP-2 and C-Kit in thyroid neoplasms. J Adv Med Med Res.

[29] Abu-Seadah SS, Attiah SM, Ali MY, Shams El-Din M, El-Kholy MA (2023). Immunohistochemical expression of HBME-1 and TROP-2 in some follicular-derived thyroid lesions. Asian Pac J Cancer Prev.

